# HealthCARE Principles: A Model for Healthy City Collaboratives

**Published:** 2007-03-15

**Authors:** James E Bailey, David M Mirvis, Charles M Key, Richard L Kyte, Michael J McCord

**Affiliations:** Division of General Internal Medicine, University of Tennessee Health Science Center, Departments of Medicine and Preventive Medicine, Memphis, Tenn; Center for Health Services Research, Department of Preventive Medicine, University of Tennessee Health Science Center, Memphis, Tenn; University of Memphis, Memphis, Tenn; Reinhart Institute for Ethics in Leadership, Viterbo University, LaCrosse, Wis; The MicroInsurance Centre, Stockbridge, Wis

## The Need for Health Care Principles

Community health-improvement collaboratives, which represent both health care consumers and health care providers in efforts to improve health care systems at the local level, are becoming a major force for improving health care systems throughout the world (1-3). However, many authors have argued that members of local collaboratives must unite around shared principles in order for their efforts to be successful (4-7). This article describes the development of a set of ethical principles, based on essential health needs, that can serve as a common foundation for collaboratives attempting to improve local health care systems.

Many nations have already organized their health care systems according to principles chosen to help them best meet the needs of consumers. For example, Canada based its health care system on the principles of comprehensiveness, universality, portability, accessibility, and public administration (8). Similarly, the proposed Clinton health plan (9) and Newt Gingrich's recommendations for transforming the U.S. health care system (10) both placed basic ethical principles and fundamental consumer health interests at the forefront.

## Development of the Memphis HealthCARE Principles

In early 2000, as a small group of community leaders in Memphis, Tennessee, considered how to reorganize regional health systems to better meet the needs of their community, they sought to articulate principles that communities could use to improve the health of community members. These leaders served as the founding board for a newly incorporated nonprofit Memphis health-improvement collaborative that was to become the Healthy Memphis Common Table. The founding board's first step was to form a diverse, 12-member interdisciplinary team that included the 9 founding board members and 3 additional community representatives. The board consisted of four experts in pertinent areas (health care policy, preventive medicine, international health insurance finance, and ethics), three consumer representatives (a small business owner, a person with a chronic illness, and a faith community representative), a primary care physician, and a specialist physician. The three additional members added to the interdisciplinary team were an attorney with expertise in corporate health care, a political scientist, and a nurse. This team led a 5-year process to identify the principles that can best guide health care providers, payers, and consumers toward common goals related to the health of community members and to the quality of the health care that they receive (Table 1).

Team members began by brainstorming at a group retreat during which they produced a preliminary list of potential core principles. They then conducted independent literature reviews to identify ethical principles articulated by other health care systems and shared their findings with all team members. The group next identified a list of core ethical principles that other systems had in common and merged this with the preliminary list. Team leaders then refined this augmented list of principles with facilitator assistance. During near monthly meetings, the team continued to refine its list of principles through a consensus process until team members reached agreement on what the principles should be and how they should be worded.

The principles identified during this process were adopted as the founding principles of the Healthy Memphis Common Table, a healthy city collaborative for the Memphis metropolitan area (11-13). In November 2003, the Healthy Memphis Common Table organized a summit at which it presented the principles to community leaders. At the end of the summit, in a public ceremony attended by more than 300 health care leaders, the chief executive officers of all the major area hospitals, together with government, public health, physician, consumer, and faith community leaders, publicly signed a pledge to uphold the principles.

Following the initial publication of the principles, the Healthy Memphis Common Table board conducted a second group consensus process to consider additional public input and formulate an acronym for these principles that would be useful in disseminating them to the public. The acronym they came up with, HealthCARE (*health* plus *choice, access, responsibility,* and *education* in health care), depicts the health care principles shared by health care consumers, providers, and payers (Table 2). These principles provide a framework for bringing everyone together in a spirit of cooperation around a "common table" to improve the health and health care of the community.

## The Memphis HealthCARE Principles

The following principles are based on what people need from a health care system in order to flourish. The broad acceptance of such needs-based principles requires that community members share a basic conception of what minimum standards for human health and health care will be sufficient to enable them to pursue happiness without outstripping their community's ability to provide what are determined to be necessary services.

### Health

The principle of *health* means that all constituents of a health care system must commit to making the health of community members their first priority. Health care providers or systems that put financial profit, shareholder interest, or political gain ahead of patients' health are less likely to truly serve individual and community needs, as are not-for-profit systems that place financial, research, educational, or other interests ahead of their patients' health. The health principle demands that all health care systems inform their partners or shareholders that their first responsibility is to serve their patients and that they make themselves transparently accountable to this standard through public reporting of their performance data.

The health principle further affirms that people need health, not simply health care services. A corollary of this principle is that the health care industry must redefine health care to include everything that people need to be healthy. Health care systems should expand beyond the bounds of hospitals, clinics, and traditional public health activities and consider all factors that affect people's health, including their economic condition, their occupation, their education, their behavior, and their environmental exposures. Communities, particularly in developing nations, frequently need to consider these factors first when working to improve the health of community residents.

### Choice

The *choice* principle derives from the ethical principle of autonomy, which recognizes the fundamental nature of free choice and self-determination. Respect for a person's freedom to choose directly reflects Immanuel Kant's most fundamental moral principle, that people should not be treated merely as a means to advance another person's self-interest (14). The choice principle is also consistent with the World Health Organization's Alma-Ata declaration following the International Conference on Primary Health Care in 1978, which included the statement, "The people have the right and duty to participate individually and collectively in the planning and implementation of their health care" (15). The choice principle means that people should participate not only as payers but also as partners in pursuing optimal health.

This principle does not imply that choice is only possible in independent fee-for-service systems, nor does it require that people be offered an infinite choice of insurance benefit options, providers, or treatments. However, it does reflect consumers' desire for some choice of providers and treatment options, and well-designed health plans with sufficiently diverse provider panels should be able to offer them such options. Studies have shown that a choice of insurers, health plans, and benefit packages may be substantially less important to consumers than having accessible, high-quality health care (16,17).

### Access

The *access* principle is based on the premise that access to health care is a fundamental good that all just health care systems should work to ensure. The Alma-Ata declaration recognizes that a just community has a basic responsibility to provide community members with universal access to primary health care. To achieve such universal health care access, the members of a society must accept that they have a duty to ensure that all members of their society receive primary health care.

### Responsibility

The principle of *responsibility* is based on the premise that people need to take personal responsibility for their own health but are also obligated to care for their neighbors by helping them to obtain services that promote health. Consumers, providers, and health care institutions must all take responsibility for the health of community members and for the use of the health care resources with which they are entrusted. All of the world's major religions recognize the importance of hospitality ― the responsibility of people to care for one another and especially the responsibility of the "host" toward his or her "guest." Indeed, this responsibility of a host to be hospitable is inherent in the name hospital.

### Education 

The principle of *education* reflects the responsibility of healthy community collaboratives to encourage all their partners, including both health care providers and health care consumers, to continually strive to learn and to share what they learn with others. Devotion to evidence-based, cost-effective care is essential to the improvement of health care systems. As Mintzberg noted in an article on the management of government programs, everyone in a health care organization designed for public benefit should serve as 1) a worker in the organization, 2) a citizen with a right to expect needed care, and 3) an informed customer whose demand for quality helps to create a marketplace that provides exceptional value in health care (18).

The five HealthCARE principles described here are interdependent and sometimes in conflict. For example, the principle of responsibility requires that consideration be given toward using resources in a way that best meets population needs or the common good, whereas the principle of choice requires that consideration be given to the personal needs and desires of individuals within that population. Communities thus may sometimes need to balance the demands of competing principles, in this example, perhaps by limiting the health care choices of community members to those that value-conscious community members might reasonably expect. Decisions that are best for a community are those that reflect both individual and population needs (7).

## Community Validation of the HealthCARE Principles

Since affirming the HealthCARE principles, the community partners of the Healthy Memphis Common Table have worked together to launch a community-wide obesity and diabetes initiative. This effort has been directed by a community partners council that includes representatives from health care consumers, small and corporate businesses, government entities, schools, hospitals, health care providers, insurance companies and health plans, quality improvement organizations, universities, trade groups, media, fitness centers, youth groups, faith-based organizations, not-for-profit agencies, and medical advocacy and support groups (19). Several competing major providers are working together for the first time on this initiative, and more than 15 multidisciplinary action teams are working through community partner organizations to build community awareness about the health risks of diabetes and obesity, as well as to augment community screening efforts, improve obesity and diabetes management, and demonstrate a business case for health promotion efforts (13). The Healthy Memphis Common Table is also expanding current efforts to improve chronic disease care in the Mid-South region of the United States by publicly reporting information on the quality of health care and by engaging consumers and providers in efforts to improve the quality of care. These efforts are now part of the Robert Wood Johnson Foundation's national Aligning Forces for Quality program to engage consumers, physicians, and payers in improving health care quality (20).

Health care principles, such as those of HealthCARE, provide a framework on which communities can base their expectations for justice in health care and develop health care systems that are accountable to community members and committed to the good of society. By rallying communities around common goals, healthy city collaboratives can help improve local health care systems, but to be most effective and overcome divisions that afflict the health of our communities, these collaboratives must foster broad participation and consensus among community members. Because of the local nature of many health issues, communities should adopt, affirm, and adhere to health care principles that hold all community members ― consumers, providers, health care administrators, insurers, businesses, government entities, and other institutions ― accountable for the health of people in their own neighborhoods.

## Figures and Tables

**Table 1:** How the HealthCARE Principles Were Developed

Founding members of the Memphis health-improvement collaborative brought together a 12-member interdisciplinary team that included philosophers, political scientists, and ethicists to identify fundamental principles of ethical health care.
The interdisciplinary team reviewed principles of existing collaboratives and national health care systems and proposals for reforming health care systems.
The interdisciplinary team gathered iterative feedback from national experts to: Identify common elements in existing statements of principles by other collaboratives or health care systems Reach a consensus on what the fundamental principles should be Draft and refine the precise wording of these principles
The team published and disseminated the principles and solicited further public comment and feedback about them.
The principles were adopted and publicly affirmed by more than 100 community partner organizations and more than 300 community leaders at the first Healthy Memphis Common Table Summit.
The board of the Healthy Memphis Common Table conducted a second group consensus process with iterative review to organize the principles into a recognizable and easily disseminated acronym: HealthCARE.

**Table 2:** HealthCARE Principles

*Health:* We seek to reorganize our health system to promote health as its primary goal.
*Choice:* We choose the best value in health care providers and treatments.
*Access:* We provide care according to need for all people.
*Responsibility:* We take responsibility for our health and are accountable for our health care resources.
*Education:* We continually learn and share how to improve our health and the health care system.
